# H_2_O_2_-responsive VEGF/NGF gene co-delivery nano-system achieves stable vascularization in ischemic hindlimbs

**DOI:** 10.1186/s12951-022-01328-6

**Published:** 2022-03-19

**Authors:** Youlu Chen, Zuoguan Chen, Jianwei Duan, Liang Gui, Huiyang Li, Xiaoyu Liang, Xinxin Tian, Kaijing Liu, Yongjun Li, Jing Yang

**Affiliations:** 1 Tianjin Key Laboratory of Biomaterial Research, Institute of Biomedical Engineering, Chinese Academy of Medical Science and Peking Union Medical College, 236 Baidi Road, Tianjin, 300192 People’s Republic of China; 2 Department of Vascular Surgery, Beijing Hospital, National Center of Gerontology, Chinese Academy of Medical Science and Peking Union Medical College, 1 Dahua Road, Beijing, 100730 People’s Republic of China

**Keywords:** Vascular endothelial growth factor, Nerve growth factor, Gene therapy, Hydrogen peroxide, Angiogenesis

## Abstract

Peripheral vascular disease (PVD) is a common clinical manifestation of atherosclerosis. Vascular endothelial growth factor (VEGF) gene therapy is a promising approach for PVD treatment. However, due to single-gene therapy limitations and high H_2_O_2_ pathological microenvironment, VEGF gene therapy are not as expectations and its clinical application are limited. Synergistic effects of Nerve factors and vascular factors in angiogenesis have attracted attention in recent years. In this study, VEGF and nerve growth factor (NGF) genes co-delivery nanoparticles (VEGF/NGF-NPs) were prepared by using H_2_O_2_ responsive 6s-PLGA-Po-PEG as a carrier. 6s-PLGA-Po-PEG could react with H_2_O_2_ specifically due to the internal peroxalate bond. Angiogenic effects of VEGF/NGF-NPs has been evaluated in cells and hindlimb ischemia mice model. Results showed that VEGF/NGF-NPs promoted VEGF and NGF co-expression simultaneously, eliminated excessive H_2_O_2_, strengthened reactions between SH-SY5Ys and HUVECs, and finally enhanced migration, tube formation, proliferation and H_2_O_2_ damage resistance of HUVECs. VEGF/NGF-NPs also recovered blood perfusion, promoted the expression of VEGF, NGF, eNOS and NO, and enhanced vascular coverage of pericytes. Treatment effects of VEGF/NGF-NPs may related to VEGF/eNOS/NO pathway. Altogether, VEGF/NGF-NPs eliminated excessive H_2_O_2_ while achieving gene co-delivery, and promoted stable angiogenesis. It’s a promising way for PVD treatment by using VEGF/NGF-NPs.

**Graphical Abstract:**

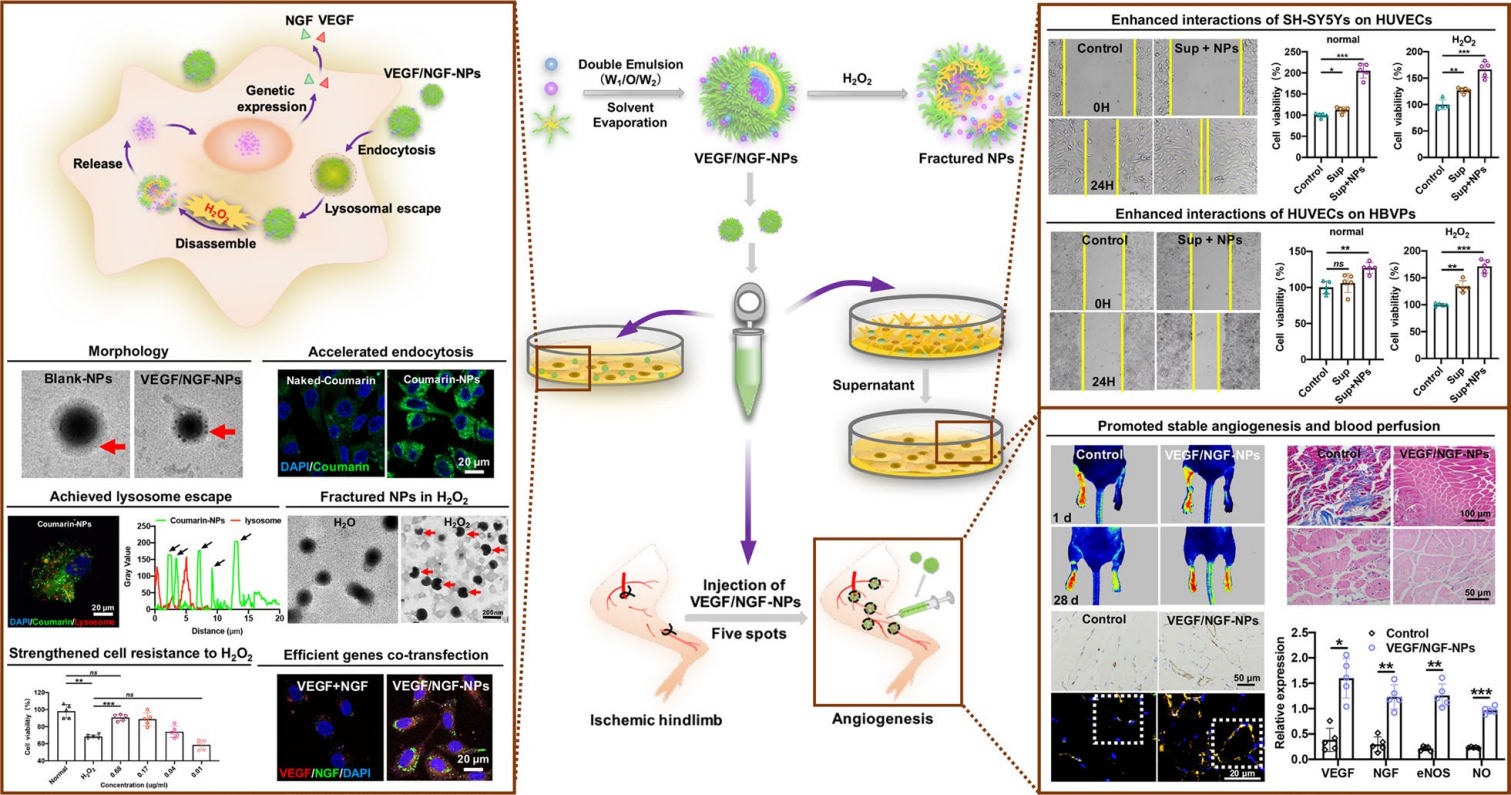

**Supplementary Information:**

The online version contains supplementary material available at 10.1186/s12951-022-01328-6.

## Introduction

Peripheral vascular disease (PVD) is one of the most common vascular diseases, which refers to non-myocardial artery obstruction or stenosis, affecting more than 200 million people worldwide. PVD often showed lower extremity blood flow damage and increased hydrogen peroxide (H_2_O_2_) content [1]. Blood vessel by-pass grafting and endovascular therapy have been regarded as "gold standard" among PVD treatments [2]. However, those surgeries require a long recovery time and may be associated with a variety of complications, such as graft infection, graft thrombosis, wound rupture or infection, and chronic lower extremity edema. In addition, there are many patients who don’t meet the treatment conditions, and 30% of them will undergo extensive amputation [3]. These problems have brought great challenges to clinical treatment of PVD.

Gene therapy is an attractive strategy to promote angiogenesis for repairing ischemic tissue perfusion. It generates new blood vessels by transferring specific genes, such as vascular endothelial growth factor gene (pVEGF) [4, 5]. VEGF is a major regulator of angiogenesis and a key factor in initiating complex cascades, which leads to the formation of new vascular networks [6]. VEGF up-regulates nitric oxide synthase (eNOS) protein level, and nitric oxide (NO) produced by eNOS significantly contributes to the prosurvival/proangiogenic program of capillary endothelium by triggering and transducing cell growth and differentiation [7–9]. However, clinical trials of pVEGF therapy have been disappointing for poor stability of neovascularization. This may be due to the limitations of single-gene therapy, which is different from natural angiogenesis. Moreover, gene transfection efficiency is poor in H_2_O_2_ microenvironment.

Angiogenesis is a complex process involving interconnections of molecular and tissue signals, in which neural interaction is essential. It has been found that nerve growth factor (NGF), as a pleiotropic factor acting on blood vessels and nerves, promotes angiogenesis and vascular repair [10]. Therefore, co-delivery of pVEGF and NGF gene (pNGF) in ischemic hindlimb may expect to simulate interactive support of blood vessels and nerves, and enhance stable therapeutic angiogenesis ultimately.

Oxidative stress and redox regulation are also key issues in the PVD. Reactive oxygen species (ROS) is regarded as a double-edged sword in regulating various cellular signal transduction processes. When produced ROS exceed natural level of cells, it was found to cause destruction of nucleic acids, proteins, lipids and other biological molecules, and eventually trigger cell death [11]. Among numerous kinds of ROS, H_2_O_2_ is the main component. Therefore, the use of H_2_O_2_ scavenging materials is expected to construct an immune microenvironment conductive angiogenesis. In our group, PLGA and PEG were covalently linked by peroxalate bonds, which could react with H_2_O_2_ specifically [12]. While reducing excess H_2_O_2_ in pathological microenvironment, NPs could realize the responsive release of genes, thus higher gene transfection efficiency could be achieved.

In this study, pVEGF and pNGF co-delivery nanoparticles (VEGF/NGF-NPs) were prepared by using 6s-PLGA-Po-PEG as carrier. The characterization of VEGF/NGF-NPs, including cellular uptake, lysosomal escape, genes transfection, H_2_O_2_ scavenging, cell migration, tube formation and intercellular interactions, were evaluated in vitro. Therapeutic effects of VEGF/NGF-NPs in angiogenesis were also evaluated in hindlimb ischemia mice model by detecting the expression of VEGF, NGF, CD31, αSMA, NG_2_, eNOS, NO, the repair of muscle tissue and the recovery of blood perfusion in ischaemic hindlimbs.

## Materials and methods

### Materials

pVEGF and pNGF were purchased from Aibimeng Biotechnology Co., Ltd (Jiangsu, China). Protamine sulfate and coumarin 6 were obtained from Aladdin (Shanghai, China). Paraformaldehyde, DAPI, MTT, RIPA Lysis Buffer, 5 × SDS-PAGE Sample Loading Buffer, 20 × TBST buffer, ECL Western Blotting Substrate and Color Mixed Protein Marker were purchased from Solarbio (Beijing, China). SDS-PAGE gel preparation kit and Lyso-Tracker were bought from Beyotime (Jiangsu, China). Hydrogen peroxide (H_2_O_2_, 30 wt %) was purchased from Tianjin Guangfu Fine Chemical Research Institute (Tianjin, China). 2,7-dichlorodihydrofluorescein diacetate (DCFH-DA) was purchased from Sigma-Aldrich (St.Louis, MO, USA). Matrigel was purchased from Corning (New York, USA).

### Cells and animals

The human umbilical vein endothelial cells (HUVECs) and SH-SY5Ys cell line were obtained from the Cell Bank of Chinese Academy of Sciences. HBVPs were purchased from Shanghai Yubo Biotechnology Co., Ltd. Female 6-week-old ICR mice were purchased from SPF (Beijing) Biotechnology Co., Ltd. (China) (Approval No.: SCXK(Jing): 2019–0010).

### Preparation of NPs

Preparation of VEGF-NPs (or NGF-NPs): distilled water solution containing 1.5 mg protamine sulfate was slowly added into dichloromethane solution containing 80 mg 6s-PLGA-Po-PEG under ice bath condition at 20,000 rpm of homogenizer. Under same conditions, distilled water solution containing 1.5 mg of pVEGF (or pNGF) was added slowly into the above solution. Under the condition of No.3 Power of homogenizer, above solution was dropwise added into 15 mL 1% PVA solution slowly and stirred for 20 min. Then, dichloromethane was removed by stirring and volatilizing for 3 h at room temperature (RT). In this case, VEGF-NPs (NGF-NPs) were obtained.

The preparation of Blank-NPs, Coumarin-NPs and VEGF/NGF-NPs: Blank-NPs were prepared without any plasmids added; Coumarin-NPs were prepared with 8 mg coumarin 6 added to replace plasmids; VEGF/NGF-NPs were prepared by adding 1.5 mg pVEGF, 1.5 mg pNGF and 3 mg protamine sulfate. The remaining steps were the same as above.

### Characterization of NPs

Size, PDI and zeta potential of NPs were measured by zeta sizer nano ZS (Malvern instruments, UK) (n = 3). Stability of NPs within 25 d was determined by zeta sizer nano ZS every 5 d (n = 3).

Morphology of NPs: 100 μL Blank-NPs, VEGF-NPs, NGF-NPs and VEGF/NGF-NPs suspension were dropped on copper mesh. After drying overnight, the morphology of NPs were observed by transmission electron microscopy (TEM) (h-6009iv, Hitachi).

Gene’s encapsulation: Supernatant was obtained by centrifuging NPs suspension, and then agarose gel electrophoresis was used to detect whether there were unencapsulated genes in the supernatant.

Gene’s release: After 2.8 mg NPs were dissolved into 4 mL PBS (or 40 μM H_2_O_2_ solution), placed them in 37 °C constant temperature shaker. On the 1, 3, 5, 8, 12, 16, 20 and 25 d, the above solutions were centrifuged at 15,000 rpm to get supernatants. After supernatants were collected the same volume of PBS was added. The content of plasmid was detected by nucleic acid densitometer at 260 nm, and the cumulative release curve was drawn (n = 3).

$$\mathrm{Cumulative\, release }(\mathrm{\%})\hspace{0.17em}=\hspace{0.17em}\frac{\text{Cumulative release of }{\text{pDNA}}}{\text{Total mass of loaded }{\text{pDNA}}}\times 100\mathrm{\%}$$

### Celluar uptake and lysosomal escape of NPs

HUVECs (or SH-SY5Ys) were seeded into confocal dish at a density of 5 × 10^4^ cells/dish. HUVECs (or SH-SY5Ys) were incubated with 62.5 μg/mL coumarin (naked-Coumarin group) or 687.5 μg/mL Coumarin-NPs (Coumarin-NPs group) for 0, 2, 4 and 6 h respectively. After cleaning with PBS for 3 times, add 400 μL Lyso-Tracker Red to each dish, incubate for 4 h, and then clean with PBS for 3 times. After that, 500 μL 4% paraformaldehyde was added to each dish for 20 min at RT and then PBS was used to gently cleaned for 3 times. Added 300 μL DAPI dye to each dish and reacted at RT for 6 min, and then cleaned the dishes gently with PBS for 3 times. Finally, 500 μL PBS was added to each dish. Celluar uptake and lysosomal escape of Coumarin-NPs in HUVECs (or SH-SY5Ys) were observed by confocal laser scanning microscopy (CLSM 410, Zeiss, Jena, Germany).

### Cytocompatibility and H_2_O_2_ scavenging ability

Intracellular clearance of H_2_O_2_: HUVECs (or SH-SY5Ys) were seeded into a confocal dish with a density of 5 × 10^4^ cells/dish. After 24 h, HUVECs (or SH-SY5Ys) were incubated with 648.4 μg/mL Blank-NPs for 24 h. Control group was added with the same volume of serum-free medium. After that, the medium containing 10 μg/mL LPS was replaced and incubated for 2 h. The remaining H_2_O_2_ was determined with the amplex red assay. Finally, 500 μL PBS was added to observe the scavenging effect of Blank-NPs on intracellular H_2_O_2_ by confocal laser scanning microscopy (CLSM 410, Zeiss, Jena, Germany).

Response of NPs to H_2_O_2_ in vitro: After mixing 52.6 mg Blank-NPs with 10 μM H_2_O_2_ medium (or PBS) for 5 min, the mixed liquid was dripped onto copper mesh. Dried overnight at RT, the morphological changes were observed under TEM (h-6009iv, Hitachi). The particle size, zeta potential of NPs was measured by zeta sizer nano ZS (Malvern instruments, UK).

Cytocompatibility of Blank-NPs on HUVECs and SH-SY5Ys: HUVECs (or SH-SY5Ys) were seeded into 96-well plates at a density of 5 × 10^3^ cells/well. After 24 h of culture, HUVECs (or SH-SY5Ys) were incubated with 0.04, 0.17, 0.68, 2.70, 10.81, 43.23, 172.92 μg/mL Blank-NPs for 24 h. Control group didn’t add Blank-NPs. Then, 5 mg/mL MTT was added to each well. After 4 h, supernatant was discarded and 150 μL DMSO was added to each well. The cell plates vibrated for 5 min and the OD value of each well was detected at the wavelength of 490 nm by a Varioskan Flash microplate reader (Thermo Fisher Scientific, USA).

$$\mathrm{Cell \,viability \%}\hspace{0.17em}=\hspace{0.17em}\frac{\mathrm{OD \,value \,of \,Blank}-\mathrm{NPs\, group}}{\mathrm{OD\, value \,of \,Control \,group}}\times 100\mathrm{\%}$$

Protective effect of Blank-NPs on HUVECs in H_2_O_2_ solution: HUVECs were seeded into 96-well plates at a density of 5 × 10^3^ cells/well. After 24 h of culture, HUVECs were incubated with 10 μM H_2_O_2_ medium containing 0, 0.68, 0.17, 0.04, 0.01 μg/mL Blank-NPs for 24 h. Control group was added with the same volume of serum-free medium. Then, 5 mg/mL MTT 20 μL was added to each well. After 4 h, supernatant was discarded and 150 μL DMSO was added to each well. The cell plates vibrated for 5 min and the OD value of each well was detected at the wavelength of 490 nm by a Varioskan Flash microplate reader (Thermo Fisher Scientific, USA).

$$\mathrm{Cell\, viability \%}\hspace{0.17em}=\hspace{0.17em}\frac{\mathrm{OD\, value\, of\, H}2\mathrm{O}2\mathrm{ group}}{\mathrm{OD \,value\, of\, Control\, group}}\times 100\mathrm{\%}$$

### Gene transfection of NPs

HUVECs were seeded into confocal dishes at a density of 5 × 10^4^ cells/well for 24 h. HUVECs were then incubated with 2.68 mg/mL NPs and 96.8 μg/mL naked pVEGF or/and pNGF respectively for 48 h, and Control group was incubated with the same volume of serum-free medium. After 3 times of PBS cleaning, 500 μL 4% paraformaldehyde was added to each dish at RT for 20 min. After cleaning with PBS for 3 times, 300 μL DAPI was added to each dish. After reacting at RT for 6 min, PBS was used to clean for 3 times. Finally, 500 μL PBS was added to observe the gene transfection results by confocal laser scanning microscopy (CLSM 410, Zeiss, Jena, Germany).

### Cell migration and tube formation

Cell migration: HUVECs (or HBVPs) were seeded into 6-well plates at a density of 3 × 10^5^ cells/well, and cultured for 24 h to achieve 100% fusion. Use pipette tip to make scratch wound. Wash with PBS to remove the cells under scratch. HUVECs (or HBVPs) were incubated with 0.67 mg/mL NPs and 24.2 μg/mL naked pVEGF or/and pNGF respectively for 48 h, and Control group was incubated with the same volume of serum-free medium. Cell migration was observed and photographed under inverted microscope at 12 h and 24 h respectively (n = 4) and analysed by ImageJ software (National Institutes of Health, Bethesda, MD).

$$\mathrm{Cell \,migration }(\mathrm{\%}) =\frac{\mathrm{Initial\, scratch\, area}-\mathrm{Scratch \,area\, after\, cell\, migration}}{\mathrm{Initial\, scratch\, area}}\times 100\mathrm{\%}$$

Tube fomation: On the ice box, Matrigel was diluted with serum-free medium, and about 150 μL of Matrigel was added into each 24-well plate. The plate then placed into 37 °C incubator for 30 min to solidify the matrix. HUVECs was digested with 0.25% trypsin, and then added into serum-free medium containing 0.67 mg/mL NPs or 24.2 μg/mL naked pVEGF or/and pNGF to prepare single cell suspension of 5 × 10^4^ cells/mL. Control group cells were added with equal volume of serum-free medium. Then, HUVECs suspension was added into plate with matrix. After cultured in 37 °C incubator for 6 h, tubule formation was observed under inverted microscope and photographed (n = 4) and analysed by ImageJ software (National Institutes of Health, Bethesda, MD).

### Enhanced interaction between cells

SH-SY5Ys (or HUVECs) were seeded into 6-well plates at a density of 3 × 10^5^ cells/well, and cultured for 24 h. SH-SY5Ys (or HUVECs) were then incubated with 2.68 mg/mL VEGF/NGF-NPs or Serum-free medium for 24 h. After the incubation, supernatant was taken and centrifuged at 4000 rpm for 10 min to get the SH-SY5Ys (or HUVECs) culture supernatant (Sup) and the SH-SY5Ys (or HUVECs) culture supernatant after VEGF/NGF-NPs incubation (Sup + NPs). On one hand, HUVECs (or HBVPs) were incubated in serum-free medium or 10 μM H_2_O_2_ medium with Sup or Sup + NPs for 24 h, and Control group was incubated with the same volume of serum-free medium to test cell proliferation ability followed the method in 2.6. On the other hand, HUVECs (or HBVPs) were incubated with Sup or Sup + NPs for 12 h and 24 h, and Control group was incubated with the same volume of serum-free medium to test cell migration ability Followed the method in 2.8.

### Blood perfusion and histological detection of hindlimbs

Female ICR mice (6-week-old) were divided into 8 groups randomly: Control group (PBS), VEGF group (naked VEGF), NGF group, VEGF + NGF group (naked NGF), Blank-NPs group, VEGF-NPs group, NGF-NPs group and VEGF/NGF-NPs group. Mice were anesthetized by intraperitoneal injection of chloral hydrate (400 mg/kg) and the femoral artery of mice was excised from inguinal ligament to bifurcation of saphenous artery and popliteal artery under general anesthesia. NPs (0.67 mg/mL, 80 μL), VEGF and/or NGF plasmids (24.2 μg/mL, 80 μL) and PBS were intramuscularly injected into the ischaemic hindlimb on 1st, 3rd, and 5th day for a total of three treatments. On the 1st, 4th, 7th, 14th, 21st and 28th day after surgery, the blood perfusion of hindlimbs was monitored by Laser Doppler Flowmeter (PERIMED AB, Sweden) and results were analyzed by PIMSOFT software (PERIMED AB, Sweden) (n = 5). After sacrifice of mice, ischemic muscle was detected by Haematoxylin and Eosin (H&E) staining and Masson staining. Angiogenesis was detected by CD31 immunohistochemistry and vascular stability was detected by CD31, NG2, α-SMA, DAPI immunofluorescence staining.

### VEGF, NGF, eNOS and NO expression assay

After homogenized in 400 µL of lysis buffer, the lysates of ischaemic tissues were centrifuged at 10,000 rpm for 15 min. Equal amounts of total proteins (15 µg) collected from supernatants were electrophoresed on 10% polyacrylamide gels. Then, proteins were transferred to PVDF membranes. After that, PVDF membranes were incubated with VEGF, NGF and eNOS antibodies followed by a HRP-conjugated anti-goat secondary antibody. Super Signal Ultra chemiluminescent reagent (Pierce, Rockford, IL) was used to detecte protein immunoblot signals. The expression level of the VEGF, NGF and eNOS protein was quantified with ImageJ software (n = 5). NO was detected by NO ELISA Kit (Shanghai Lanpai Biotechnology Co., Ltd, China).

### Statistical analysis

Statistical significance between multiple groups were performed with one-way ANOVA. Data are expressed as the mean ± standard deviation. Differences with a value of *P* < 0.05 were considered statistically significant.

## Results

### Preparation and characterization of NPs

**Fig. 1:**
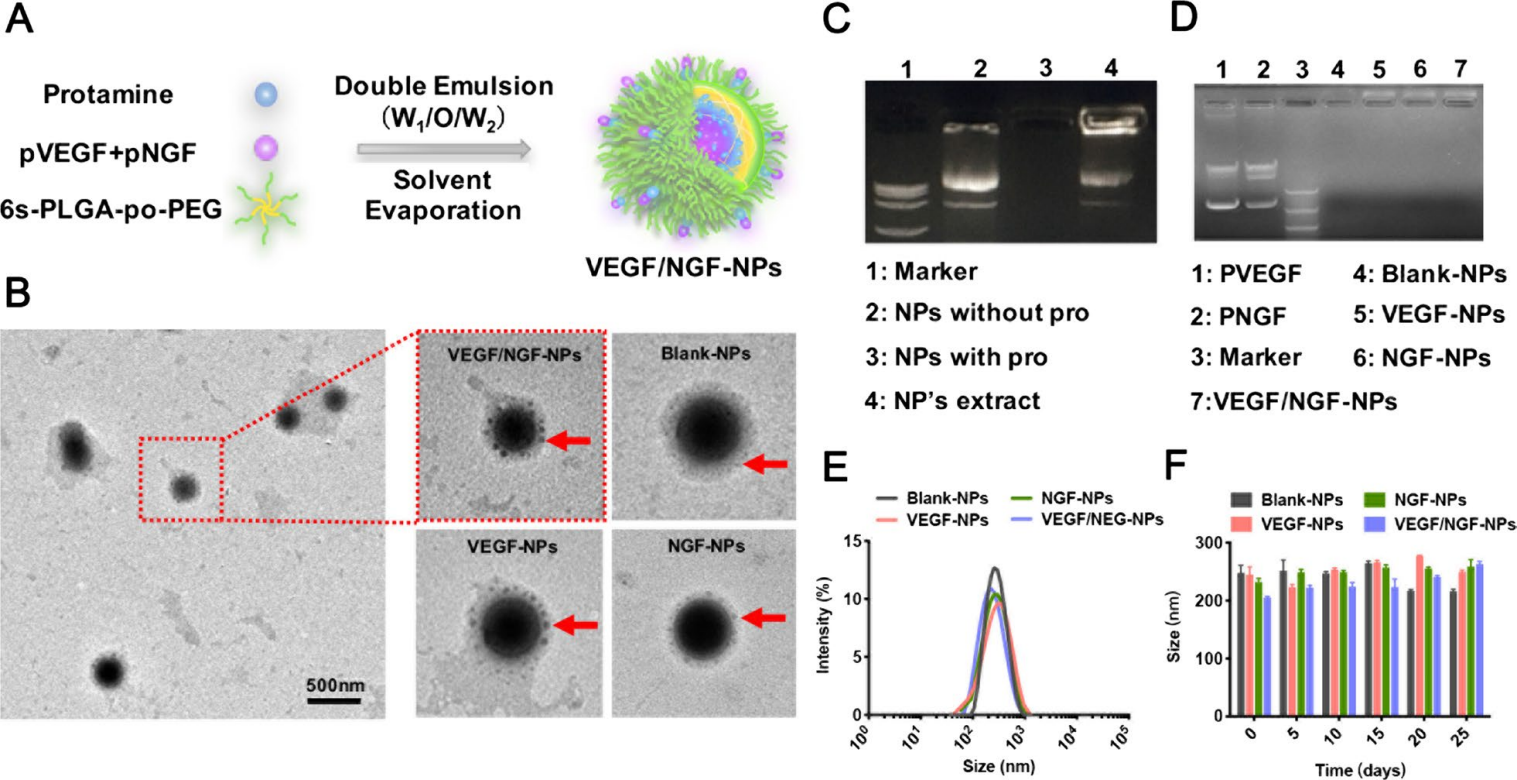
Characterization of NPs. **A** Schematic illustration of NP’s preparation. **B** Typical images of NPs under TEM. **C** Encapsulation efficiency of NPs with protamine sulfate. Without pro group stands for protamine free NPs, with pro group stands for protamine loaded NPs and NP’s extract group stands for plasmids extract after protamine loaded NPs dissolving in dichloromethane. **D** Encapsulation efficiency of gene NPs. **E** Particle size of NPs. **F** Stability of NPs within 25 days. The data are presented as mean ± SD (n = 3)

Gene NPs were prepared by high speed stirring double emulsion method (Fig. 1A), GFP plasmid (pGFP) was used as a report gene. To increase the gene loading rate in NPs, positively charged protamine was added, which could combine plasmids and NPs by electrostatic interaction. As is shown in Fig. 1B, we prepared protamine loaded GFP-NPs (with pro group) and protamine free GFP-NPs (without pro group) respectively. Meanwhile, GFP-NPs (with pro) were dissolved in dichloromethane and were extracted to release the inside plasmids with distilled water (NP’s extract group). Agarose gel electrophoresis was used to detect NP’s extracted solution and supernatant after centrifugation of NPs. It could be seen that pGFP was fully packed into NPs by adding protamine. In addition, the pGFP remained integral in the process of preparation without fragmentation. By detecting the supernatant, there were no leaked genes (Fig. 1C). Therefore, both pVEGF and pNGF were encapsulated completely.

In accordance with the foregoing method, Blank-NPs (without plasmids), VEGF-NPs (containing 1.5 mg pVEGF), NGF-NPs (containing 1.5 mg pNGF) and VEGF/NGF-NPs (containing 1.5 mg pVEGF and 1.5 mg pNGF) were prepared respectively. Morphology of NPs were observed by TEM. It could be seen that NPs were spherical with a layer of small spherical particles adsorbed on the surface. This phenomenon may be caused by the formation of complex between protamine and plasmid and adsorption on NPs surface. (Fig. 1D). Particle size, PDI and zeta potential of NPs were also detected. Particle sizes of Blank-NPs, VEGF-NPs, NGF-NPs and VEGF/NGF-NPs were (258.4 ± 7.6) nm, (244.9 ± 13.6) nm, (231.8 ± 6.5) nm and (205.2 ± 1.9) nm, respectively. PDI were 0.226 ± 0.036, 0.273 ± 0.008, 0.273 ± 0.008 and 0.217 ± 0.008, and zeta potential were − (2.70 ± 0.16) mV, − (5.03 ± 0.95) mV, − (6.70 ± 0.49) mV and − (11.27 ± 1.65) mV respectively (Fig. 1E, Additional file 1: Figure S1). In addition, NPs were stable within 25 days (Fig. 1F).

### H_2_O_2_ scavenging and pVEGF/pNGF co-transfection of NPs

Polymer-based gene delivery systems have been reported widely [13–15]. Gene delivery systems usually need to be more easily uptaken by cells, achieving lysosomal escape, releasing genes and scavenging H_2_O_2_ successfully in cells [16–18]. Therefore, H_2_O_2_ scavenge ability and gene delivery process of VEGF/NGF-NPs were explored.

**Fig. 2:**
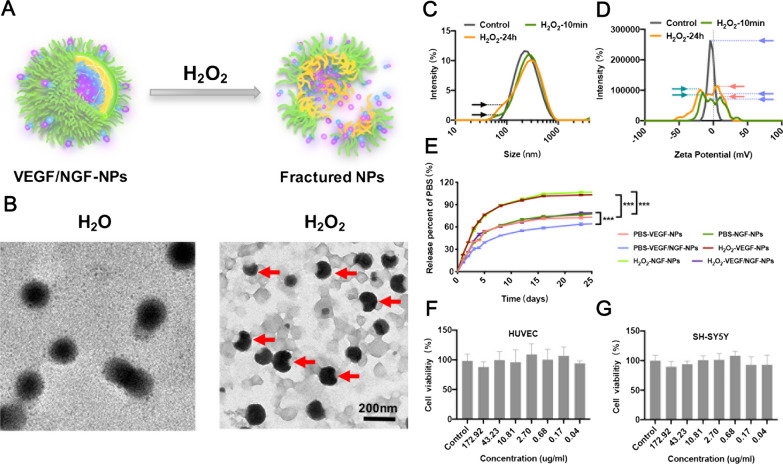
H_2_O_2_ responsiveness and biocompatibility of NPs. **A** Schematic illustration of NP’s responsiveness to H_2_O_2_. **B** Typical images of NPs in H_2_O or H_2_O_2_ under TEM. Particle size (**C**) and surface potential change (**D**) of Blank-NPs in PBS or H_2_O_2_. Genes release of NPs during 25 d in PBS or H_2_O_2_ (**E**). Biocompatibility of NPs when incubated with HUVECs (**F**) and SH-SY5Ys (**G**)

Firstly, morphology, particle size and zeta potential of NPs in H_2_O_2_ environment were observed to explore H_2_O_2_ responsiveness of NPs. Results showed that NPs was obviously broken in 10 µM H_2_O_2_ solution. Schematic diagram and representative images of TEM were shown (Fig. 2A, B). Particle size results showed that small particle fragments increased with time, proving that fragmentation of NPs was occurring continuously (Fig. 2C). Zeta potential results showed that surface charge of Blank-NPs changed from a large peak to three small peaks, including the same peak as the original peak, the peak with large negative charge and the peak with positive charge (Fig. 2D). These two new peaks may be caused by the release of protamine after NPs reacted with H_2_O_2_. In addition, gene’s release was accelerated in H_2_O_2_ environment comparing to PBS environment, indicating that gene release was intensified after NPs' broken (Fig. 2E). These results indicated that NPs was H_2_O_2_ responsive and could be broken after reacting with H_2_O_2_. In addition, Cytocompatibility of NPs was tested in HUVECs and SH-SY5Ys, and results showed that NPs had almost no toxicity to both type of cells (Fig. 2F, G).

**Fig. 3:**
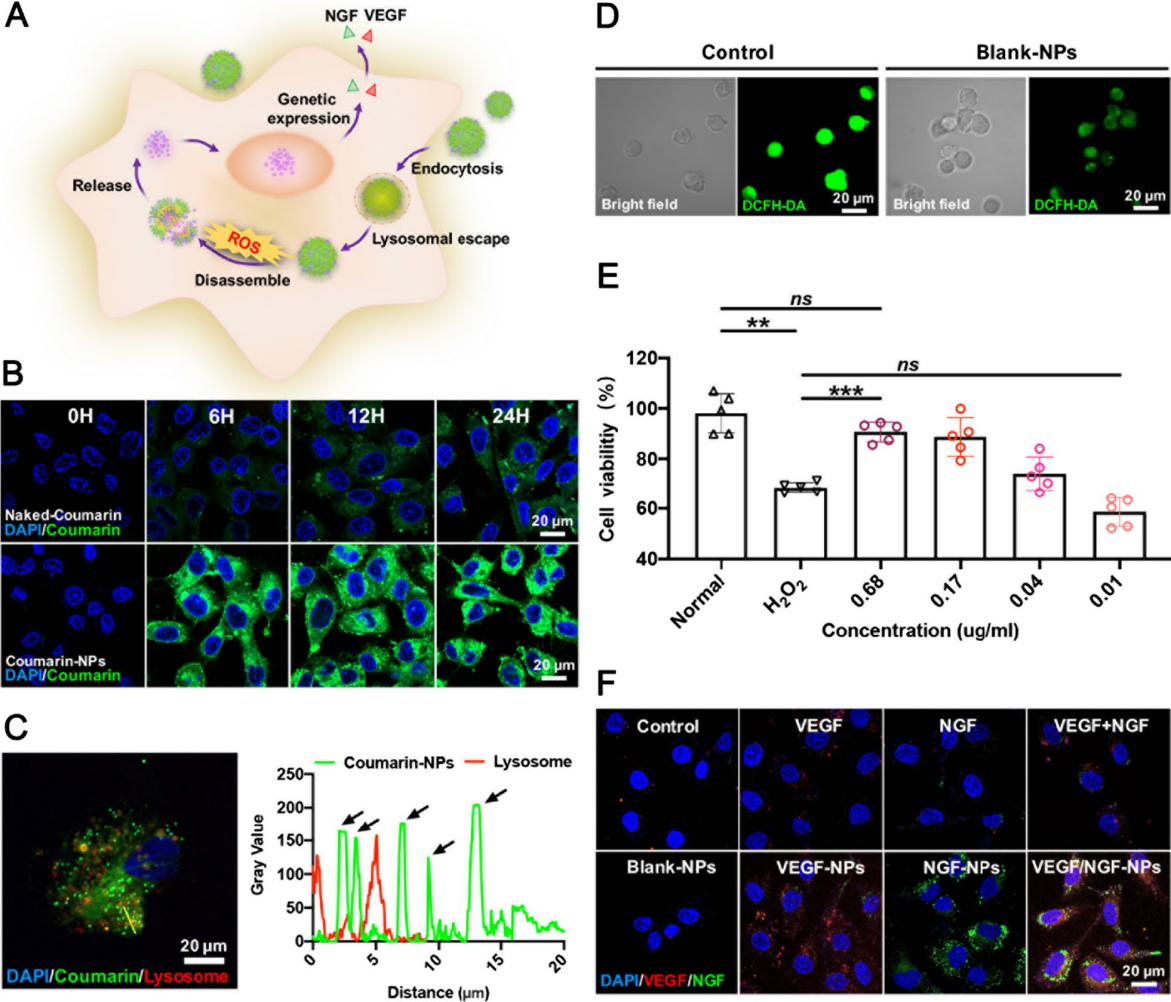
H_2_O_2_ scavenging and gene transfection of NPs in HUVECs. **A** Schematic illustration of NP’s ROS scavenging and pVEGF/pNGF co-transfection in cells. **B** Cellular uptake of naked-Coumarin and Coumarin-NPs for 0 h, 6 h, 12 h and 24 h. **C** Lysosomal escape of Coumarin-NPs. **D** H_2_O_2_ scavenging of HUVECs after incubating Blank-NPs 24 h. **E** Cell viability of HUVECs after incubation with different concentrations of Blank-NPs in 10 µM H_2_O_2_. The data are presented as mean ± SD (n = 5, ^**^P < *0.01*, ^***^P < *0.001*). **F** Gene co-transfection through NPs

Then, to explore gene delivery process of VEGF/NGF-NPs (Fig. 3A), cellular uptake, lysosomal escape, intracellular H_2_O_2_ clearance, cell protection and gene transfection of NPs were explored. Coumarin-NPs were prepared by loading coumarin 6 to observe the uptake of NPs by HUVECs. Compared with naked-Coumarin group, Coumarin-NPs could be absorbed more by HUVECs within the same amount of time (Fig. 3B), and Coumarin 6-NPs also achieved lysosomal escape successfully (Fig. 3C). H_2_O_2_ scavenging and cell protection of NPs were also detected in HUVECs. In Fig. 3D, compared with Control group, Blank-NPs attenuated ROS increasing successfully in HUVECs induced by LPS. After that, protection of NPs on HUVECs in H_2_O_2_ medium was also tested. Compared with H_2_O_2_ group, Blank-NPs resisted H_2_O_2_ injury on HUVECs effectively, and the protection was enhanced when NP’s concentration increased (Fig. 3E). Finally, gene transfection efficiency of NPs was investigated. To facilitate observation, VEGF-RFP plasmids and NGF-GFP plasmids with fluorescent protein were constructed by using PAAV-G-CMV-2A-RFP-blank and PAAV-G-CMV-2A-GFP-blank vectors (Additional file 1: Figure S2). After delivery, transfecation efficiency of NPs was observed in confocal laser scanning microscopy. Compared with free pVEGF and pNGF, delivery through NPs achieved more efficient pVEGF and pNGF transfection. More importantly, VEGF/NGF-NPs realized simultaneous and efficient co-transfection of pVEGF and pNGF (Fig. 3F).

### VEGF/NGF-NPs promoted cell migration and tubulogenesis of HUVECs

**Fig. 4:**
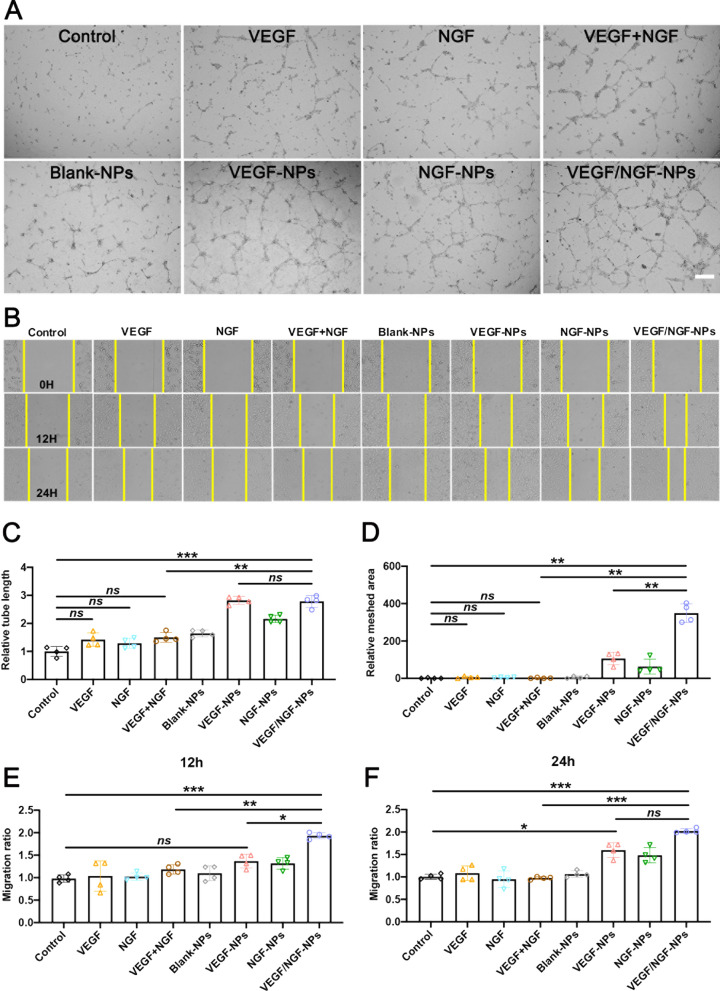
Effects of NPs on migration and tube formation in HUVECs. **A** Images of tubule formation after HUVECs incubated with NPs for 6 h was observed under inverted microscope. Relative tube length (**B**) and relative meshed area (**C**) were also quantified. **D** Migration images of after HUVECs incubated with NPs for 12 h and 24 h was observed under inverted microscope (×4). Quantification of cell migration for 12 h (**E**) and 24 h (**F**) were also quantified. The data are presented as mean ± SD (scale bar: 50 µm, n = 4, ^*^P < 0.05, ^**^P < 0.01, ^***^P < *0.001*)

Generally, gene therapy is mainly aimed at the promotion of angiogenesis and seldom pay attention to the effect on skeletal muscle remodeling in ischemic tissues. In previous studies, we found that NGF transfection could not only induce angiogenesis, but also induce the expression of type I muscle fibers in ischemic limbs [19]. Moreover, the co-transfection of VEGF gene and a second gene to enhance therapy is also considered to be a promising method to promote stable therapeutic angiogenesis in the future [20]. Therefore, in this study, we co-transfected NGF and VEGF to observe whether there is a positive synergy between them. We detected tubule formation and migration of HUVECs incubated by NPs, and representative images were given (Fig. 4A, B). Compared with Control group, VEGF group and VEGF + NGF group didn’t seem to promote tube formation or migration of HUVECs, while VEGF-NPs group and VEGF/NGF-NPs group promoted relative tube length, relative meshed area and HUVECs migration obviously. Compared with VEGF + NGF group, VEGF/NG-NPs promoted tube formation and cell migration significantly, which indicated that this gene delivery system enhanced function of its inside genes through efficient transfection. In addition, compared with VEGF-NPs group, VEGF/NGF-NPs group didn’t promote the relative tube length, but promoted the relative meshed area visibly, implying that co-delivery of pNGF assisted pVEGF in accelerating vascular network formation of HUVECs (Fig. 4C, D, Additional file 1: Figure S3 A, B). And compared with VEGF-NPs group, differences of HUVECs migration in VEGF/NGF-NPs group were little in 24 h. Co-delivery of pNGF to help pVEGF on HUVECs migration was limited (Fig. 4E, F).

### VEGF/NGF-NPs promoted intercellular interactions

**Fig. 5:**
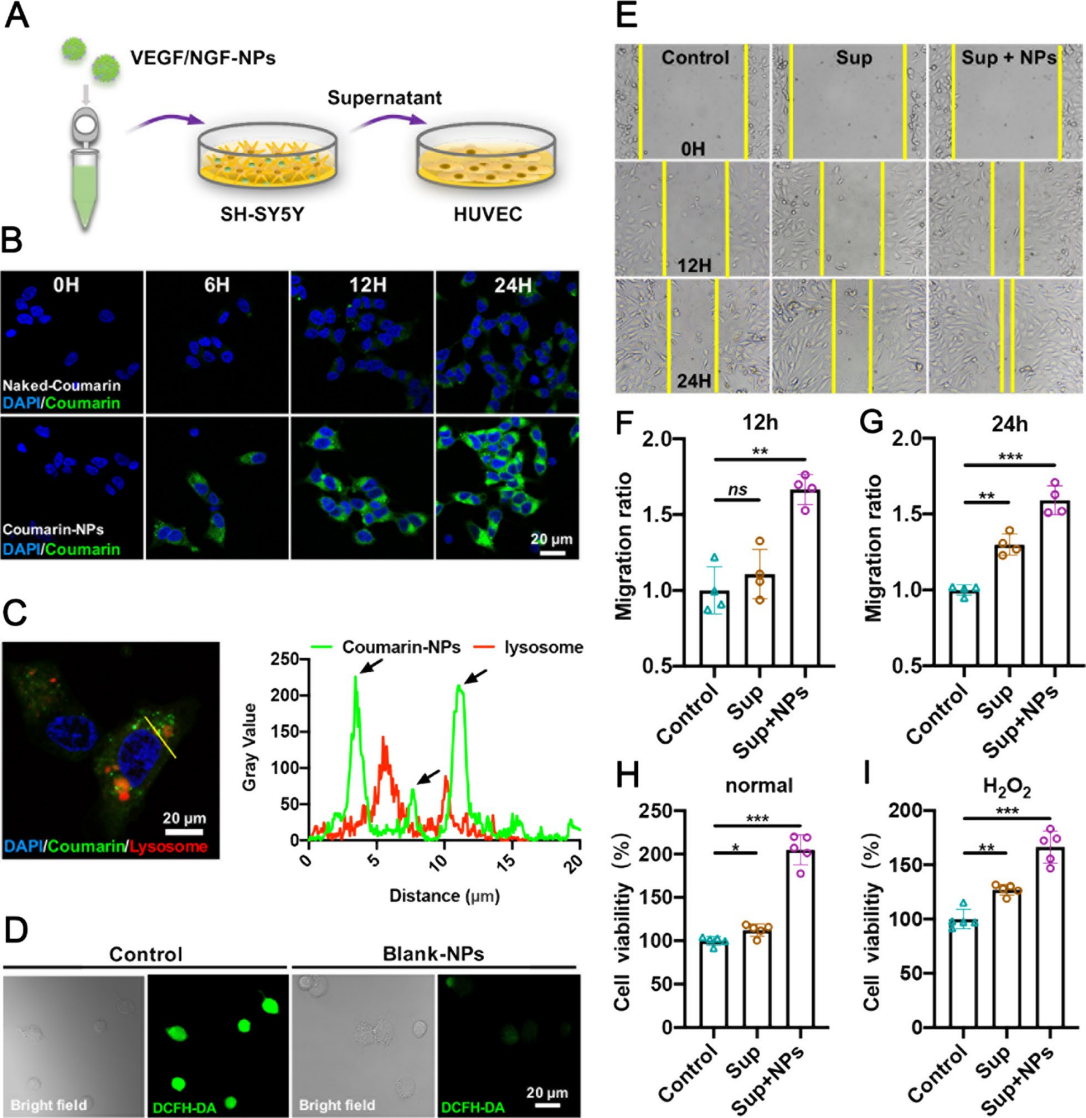
Enhanced interaction between SH-SY5Ys and HUVECs. **A** Schematic illustration of interaction between SH-SY5Ys and HUVECs. After SH-SY5Ys was incubated with serum-free medium (Control), supernatant (Sup), supernatant + VEGF/NGF-NPs (Sup + NPs) for 24 h, HUVECs were incubated with supernatant for 24 h. **B** Cellular uptake of naked-Coumarin and Coumarin-NPs in SH-SY5Ys for 0 h, 6 h, 12 h, 24 h. **C** Lysosomal escape of Coumarin-NPs in SH-SY5Ys. **D** H_2_O_2_ scavenging of SH-SY5Ys after incubating Blank-NPs in 10 µM LPS. **E** Migration images of HUVECs after incubating with serum-free medium, Sup and Sup + NPs for 12 h and 24 h. Quantification of cell migration for 12 h (**F**) and 24 h (**G**) were also quantified and the data are presented as mean ± SD (n = 4, **P < 0.01, ***P < 0.001). Cell viabilities of HUVECs after incubating with serum-free medium, Sup and Sup + NPs in PBS (**H**) and 10 µM H_2_O_2_ (**I**) and the data are presented as mean ± SD (n = 5, **P < 0.01, ***P < *0.001*)

Nutrition of nerve plays an important role in the process of ischemic tissue repair. If interactions between nerve and blood vessel enhanced, it’s hopeful to get further angiogenesis and ischemic repair [21, 22]. Therefore, we investigated whether VEGF/NGF-NPs had the potential to promote interactions between nerve and blood vessel. In this study, SH-SY5Ys were incubated with VEGF/NGF-NPs and supernatant after centrifuging (Sup + NPs) were collected in vitro, and then HUVECs were incubated with Sup + NPs to simulate the interaction process (Fig. 5A). In addition, as we all know that there are huge state differences between the same kind of cells cultured in vitro and in vivo, which made us not use the H_2_O_2_ concentration at lesion in vivo for in vitro test, but choose other suitable concentrations.

Before that experiment, we first detected cellular uptake (Fig. 5B), lysosomal escape (Fig. 5C) and H_2_O_2_ clearance of NPs in SH-SY5Ys. It could be seen that NPs achieved lysosomal escape and higher cellular uptake in SH-SY5Ys. Meanwhile, NPs also removed excessive ROS in SH-SY5Ys caused by LPS (Fig. 5D). Therefore, the gene delivery system was also feasible for SH-SY5Ys. Then, we examined the effects of Sup + NPs on migration, proliferation and H_2_O_2_ resistance of HUVECs. Firstly, we presented representative images of HUVECs migration results (Fig. 5E). Compared with Control group, Sup + NPs group promoted cell migration significantly at 12 h and 24 h, and the effects increased with time (Fig. 5F, G). Next results showed that HUVECs proliferation in Sup + NPs group was 2.05 times as much as that in Control group (Fig. 5H). Even under the influence of H_2_O_2_, HUVECs proliferation in Sup + NPs group was 1.66 times as much as that in Control group (Fig. 5I). Sup + NPs could still promote HUVECs proliferation under H_2_O_2_ injury. These results indicated that VEGF/ NGF-NPs enhanced interactions between SH-SY5Ys and HUVECs and further promoted migration, proliferation, H_2_O_2_ resistance of HUVECs markedly.

**Fig. 6:**
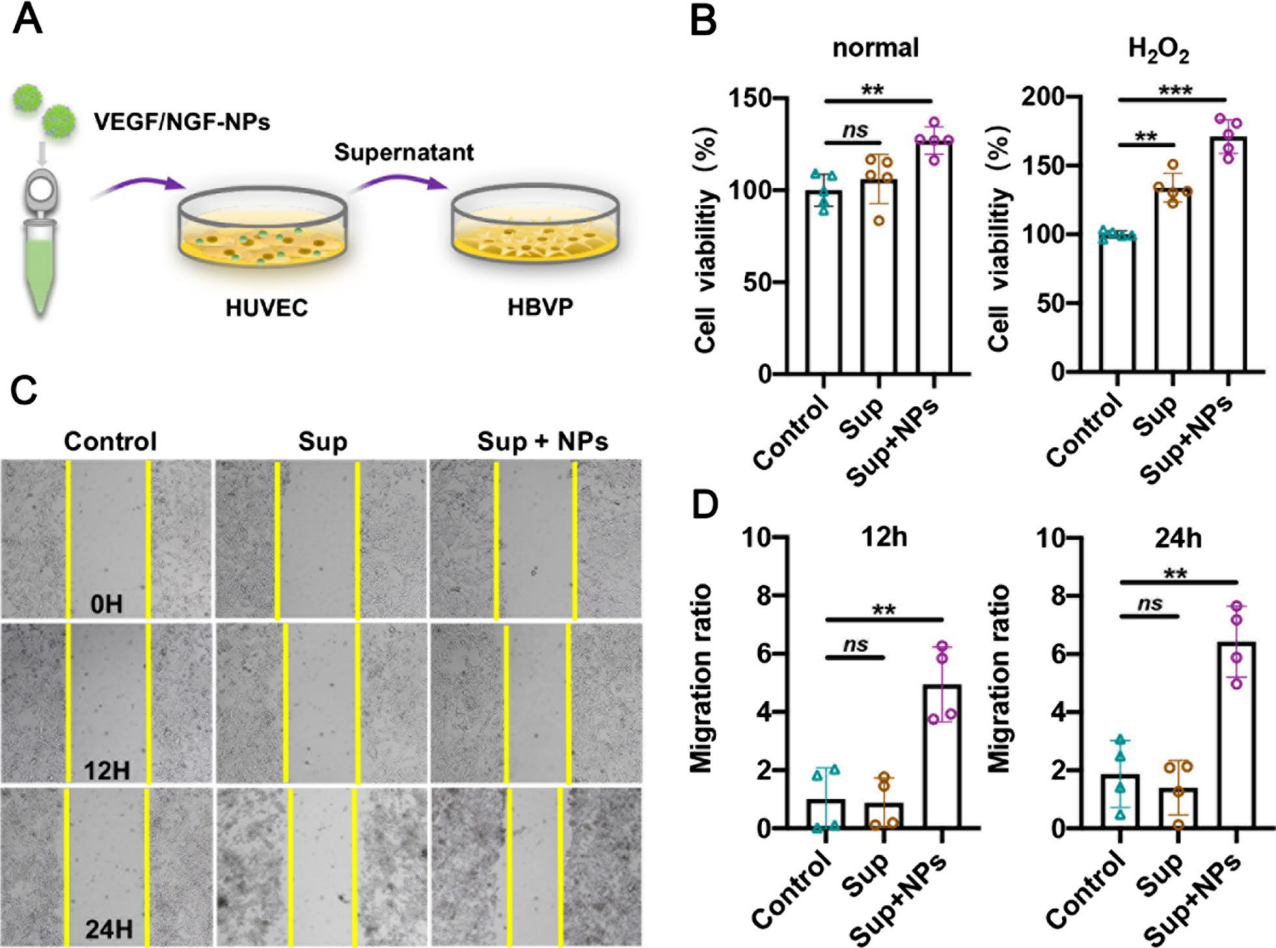
Enhanced interaction between HUVECs and HBVPs. **A** Schematic illustration of interaction between HUVECs and HBVPs. **B** Cell viabilities of HBVPs after incubating with serum-free medium, Sup and Sup + NPs in PBS and H_2_O_2_ and the data are presented as mean ± SD (n = 5, **P < 0.01, ***P < *0.001*). **C** Migration images of HBVPs after incubating with serum-free medium, Sup and Sup + NPs for 12 h and 24 h. Quantification of cell migration for 12 h (**F**) and 24 h (**D**)

Pericyte coverage is crucial to the stability of neovascularization, therefore, interactions between HUVECs and HBVPs after VEGF/NGF-NPs transfection have been also explored. HUVECs were incubated with VEGF/NGF-NPs, and centrifuged supernatant (Sup + NPs) was collected in vitro, then HBVPs were incubated with Sup + NPs to simulate interaction process (Fig. 6A). Results showed that compared with Control group, cell proliferation (Fig. 6B) and migration (Fig. 6C, D) of HBVPs in Sup + NPs group was significantly promoted, even in H_2_O_2_ environment. These results suggested that VEGF/NGF-NPs also enhanced interactions between HUVECs and HBVPs, and further promoted migration, proliferation and anti-H_2_O_2_ damage ability of HBVPs.

### VEGF/NGF-NPs promoted stable angiogenesis in ischemic hindlimbs

**Fig. 7:**
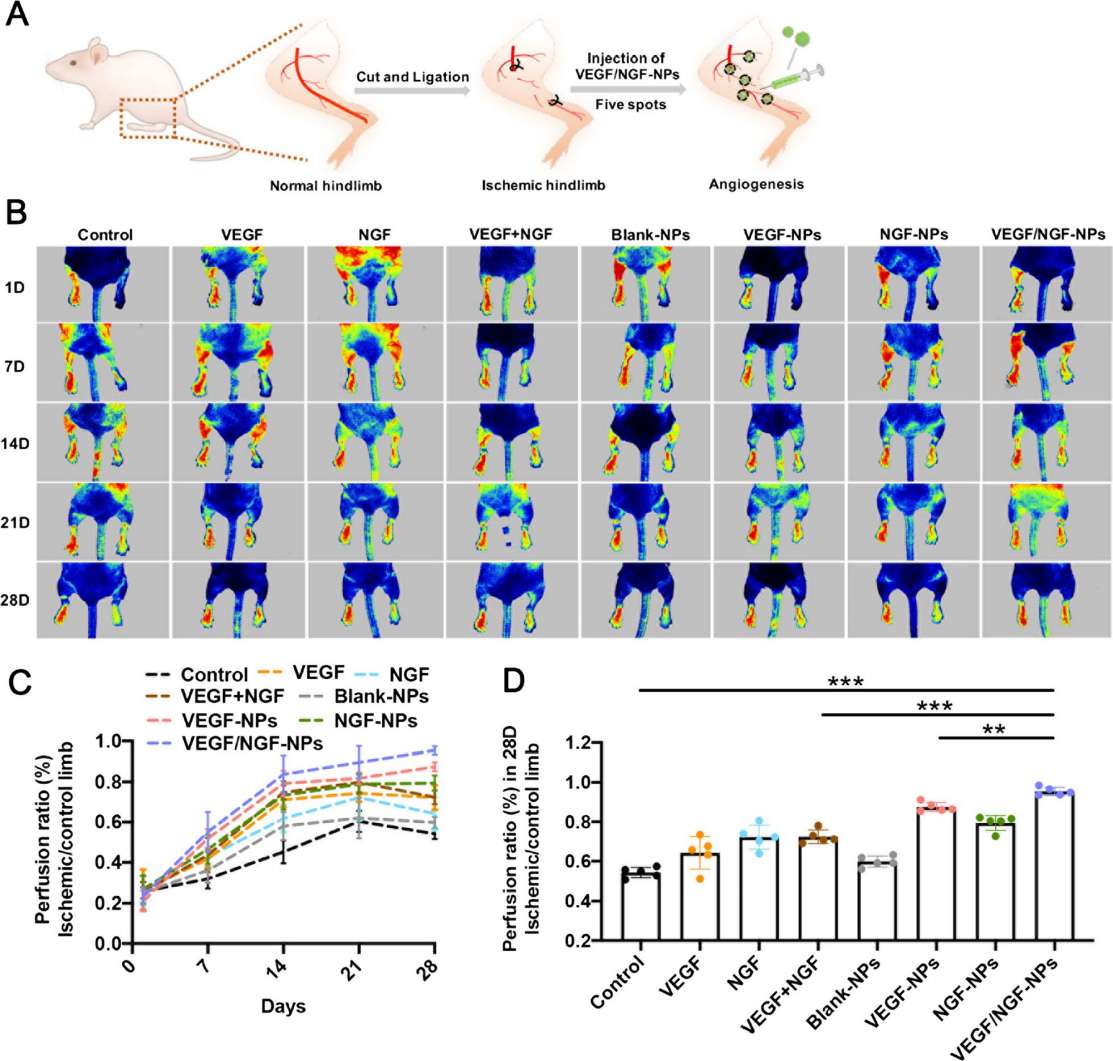
NPs affected blood reperfusion of the ischemic hindlimbs. **A** Schematic diagram of ischaemic hindlimb model construction and VEGF/NGF-NPs promoting angiogenesis. **B** Intramuscular injection of NPs to target blood flow recovery in ischaemic hindlimb was investigated on 28 days and representative laser doppler perfusion images were presented (n = 5). **C** Blood flow detected by Laser doppler perfusion at day 1, 7, 14, 21, 28 were analyzed. **D** Statistical difference of blood flow on 28 d were also showed. The data are presented as mean ± SD (n = 5, ^*^P < 0.05, ^**^P < 0.01, ^***^P < *0.001*)

**Fig. 8:**
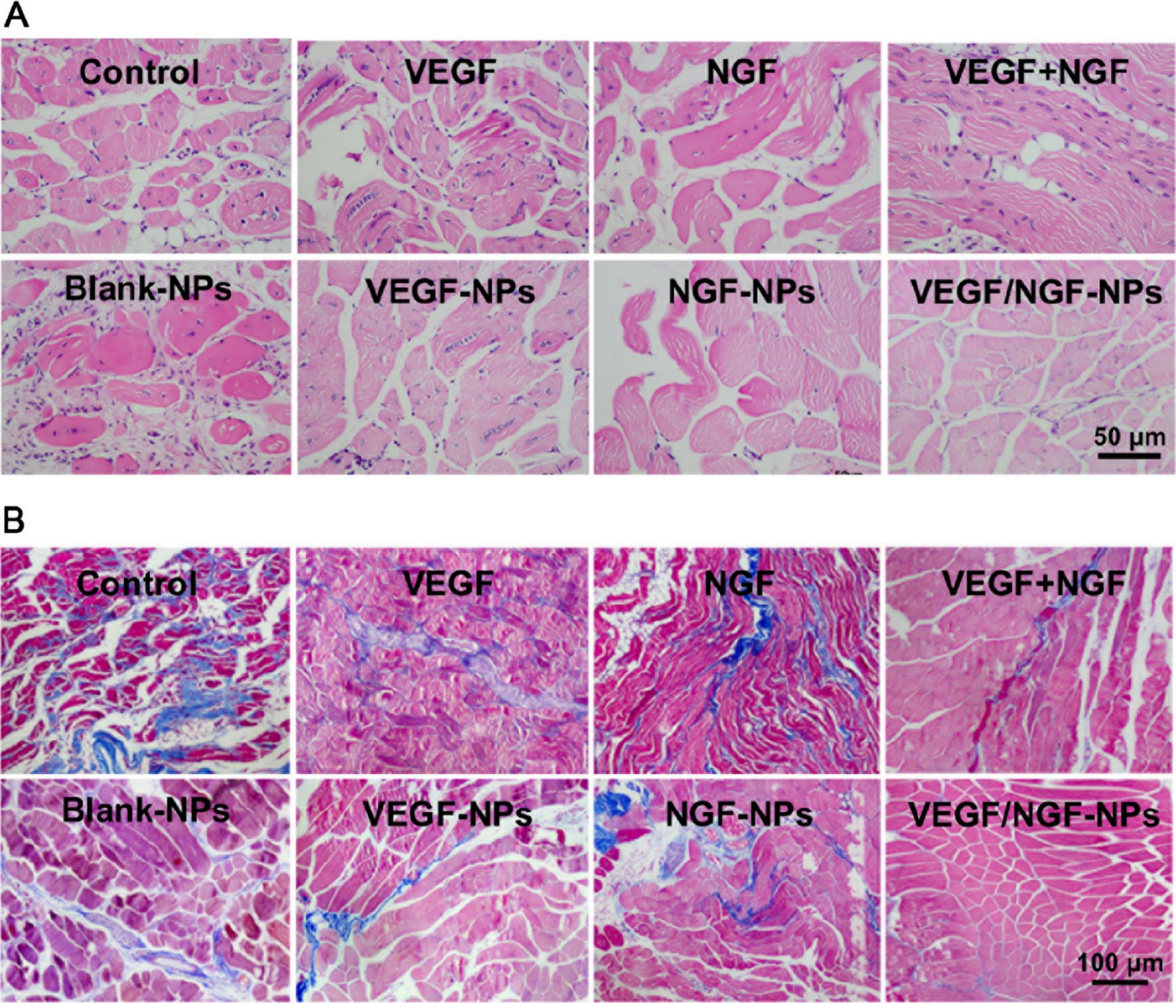
Histological evaluation of ischaemic hindlimb after treated by NPs. Haematoxylin and Eosin (H&E) staining (**A**) and Masson staining (**B**) of ischaemic hindlimb tissue treated by NPs were presented

Effects of VEGF/NGF-NPs on angiogenesis in vivo was detected. By establishing ICR mouse model of ischaemic hindlimb, we used five-point intramuscular injection of VEGF/NGF-NPs to promote angiogenesis (Fig. 7A). Figure 7B showed representative blood flow images after 28 days of ischemia. Compared to 21 days, blood flow decreased on 28 days in many groups except VEGF/NGF-NPs group, which may be due to the poor stability of neovascularization (Fig. 7C). Compared with Control group and VEGF-NPs group, blood flow of VEGF/NGF-NPs group recovered significantly on 28 days (Fig. 5D). Then H&E staining and Masson staining were performed on ischemic tissue. It could be seen that size and shape of muscle fibers in VEGF-NPs group and VEGF/NGF-NPs group were both uniform. Both VEGF-NPs and VEGF/NGF-NPs improved muscle injury and reduced the production of collagen fiber obviously (Fig. 8A, B).

**Fig. 9:**
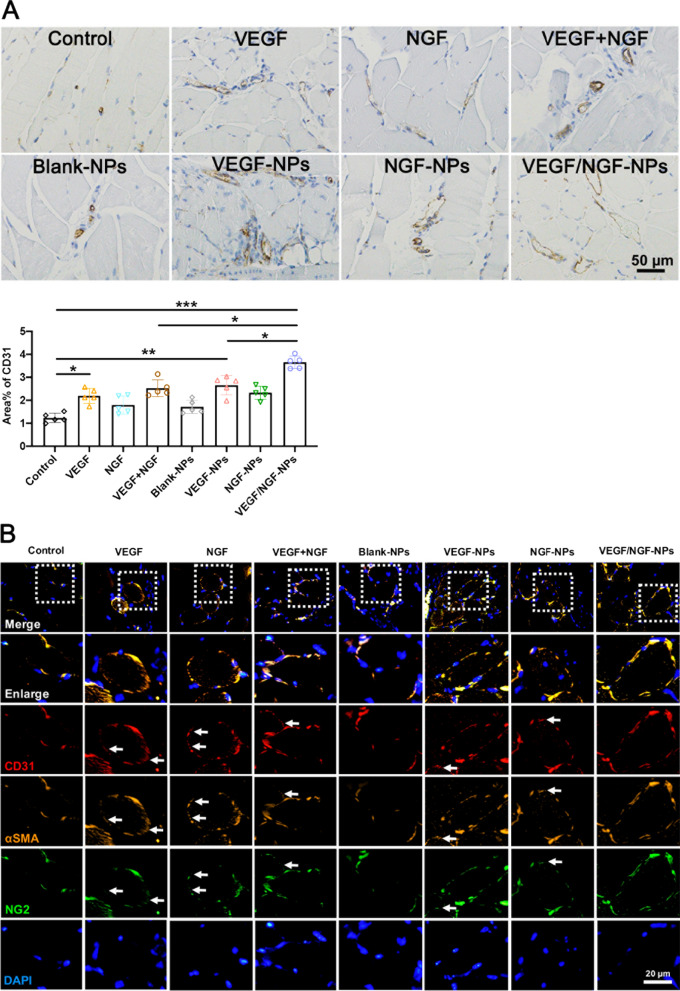
Angiogenesis and vascular stability of ischaemic hindlimb after treated by NPs. **A** Quantification of CD31-positive areas. The data are presented as mean ± SD (n = 5, *P < 0.05, **P < 0.01, ***P < *0.001*). **B** Laser confocal conpositioning representative images of CD31, α-SMA, NG2 and DAPI and are prestened

To observe the neovascularization more intuitively, we detected CD31 expression in ischemic tissues by immunohistochemistry, and representative pictures were given (Fig. 9A). Compared with Control group and VEGF-NPs group, VEGF/NGF-NPs group promoted the expression of CD31 significantly, indicating that VEGF/NGF-NPs promoted angiogenesis in ischemic tissue significantly. During this process, pNGF and pVEGF played a synergistic role. The coverage of smooth muscle cells and pericytes often reflects the maturation and stability of neovascularization. To explore neovascularization stability, we stained ischemic tissues by immunofluorescence to co-located CD31, α-SMA (marker of smooth muscle cells), DAPI and NG2 (marker of pericytes) of the vessels, and gave the representative picture (Fig. 9B). The results showed that functional vessels (10 µm–70 µm) in Control group and Blank-NPs group were less. Pericytes coverage of VEGF group, NGF group, VEGF + NGF group, VEGF-NPs group and NGF-NPs group were insufficient (white arrow) while VEGF/NGF-NPs group had relatively complete pericytes coverage. These results suggested that pNGF and pVEGF promoted stable angiogenesis in a synergistic manner.

### VEGF/NGF-NPs enhanced the expression of VEGF, NGF, eNOS and NO

**Fig. 10:**
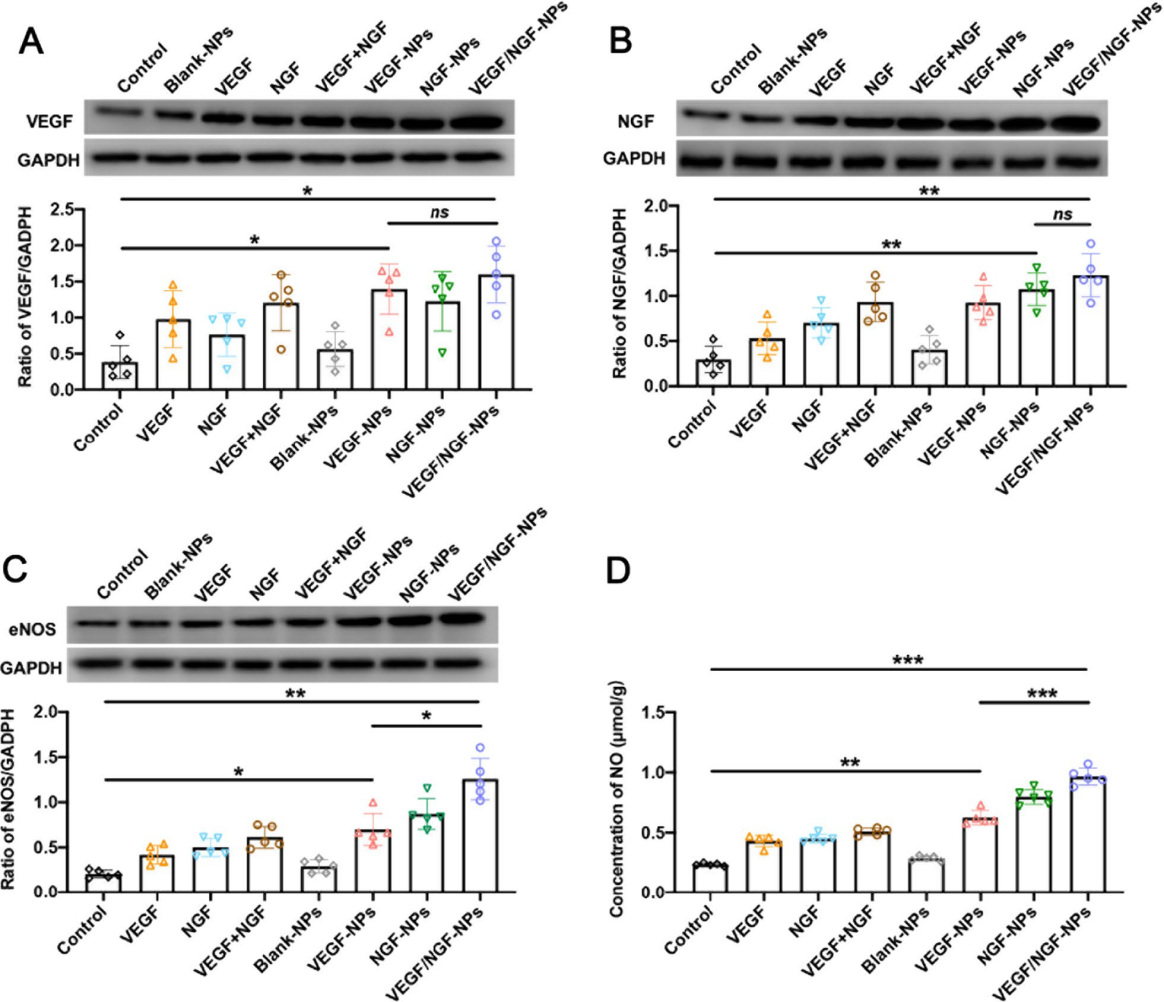
VEGF, NGF, eNOS and NO expression of ischaemic hindlimb after treated by NPs. Protein level alteration of VEGF (**A**), NGF (**B**), eNOS (**C**) was verified by western blot and NO (**D**) level alteration was verified by ELISA. All the data are presented as mean ± SD (n = 5, *P < 0.05, **P < 0.01, ***P < *0.001*)

Nitric oxide (NO) produced by endothelial cells through endothelial nitric oxide synthase (eNOS) is an important vasoactive compound [23–25]. NO is responsible for a variety of physiological and cellular processes, including endothelial cell migration, proliferation and angiogenesis. The function and activity of eNOS in endothelial cells are crucial to vascular integrity and homeostasis [26, 27]. In addition, studies have shown that NGF could improve angiogenesis through VEGF/Akt/NO dependent mechanism [28]. Therefore, expressions of VEGF (Fig. 10A), NGF (Fig. 10B), eNOS (Fig. 10C) and NO (Fig. 10D) in ischemic tissues were detected after VEGF/NGF-NPs treatment. It could be seen that VEGF/NGF-NPs enhanced the expression of VEGF, NGF, eNOS and NO in ischaemic hindlimb compared with Control group (4.16, 4.15, 6.17 and 4.13 times of Control group, respectively). It should be noted that compared with VEGF-NPs group (or NGF-NPs group), VEGF/NGF-NPs didn’t promote further expression of VEGF (or NGF) in ischemic lower limbs, but the expression of eNOS and NO was significantly promoted. This result may suggest that synergistic effect of pNGF helped pVEGF promoting more expressions of eNOS and NO.

## Discussion

Angiogenesis is an important adaptive mechanism against ischemia [29–31]. Angiogenesis through gene therapy is undoubtedly a promising method for the treatment of PVD. pVEGF therapy has brought a brief promise to people, but has failed to achieve satisfactory clinical results. Strong overexpression of VEGF by different gene therapy vectors induces the growth of abnormal hemangiomatous vascular structures in skeletal muscle. Low expression of VEGF is safe, but not sufficient to produce an effective therapeutic benefit. Appropriate VEGF angiogenic stimulation is needed, but the new vessels need to survive for a long time after the end of angiogenic stimulation to have a real therapeutic effect on PVD. Therefore, it’s particularly important to promote stable angiogenesis.

After the budding of angiogenesis, original vascular plexus is remodeled extensively. The remaining vessels are matured and stable, which marks the end of vascular plasticity [32, 33]. Vascular stability requires vascular maturation through recruitment of parietal cells (like pericytes and smooth muscle cells) and establishment of intercellular contact between parietal cells and the endothelium. In addition, pericytes have long been considering to be the main regulator of vascular maturation and stability, and the association with pericytes is necessary to make vessels independent of sustained VEGF expression [34–36]. Among many tissues and signaling molecules that influence the maturation and stability of new blood vessels, neural fiber network is an important part that cannot be ignored. Vascular circulatory system and neural fiber network have similar shape distribution and close ties, so strengthening the interactions between them is conducive to stable angiogenesis. NGF was first isolated from nervous tissue, it’s one kind of pleiotropic factor that can act on both nerves and blood vessels. Studies have shown that NGF stimulation increased the expression of Notch1 in a dose-dependent manner, which activated the Notch signaling pathway. And Notch signaling pathway plays a key role in pericytes survival [37–40]. However, it’s unknown whether NGF promotes stable angiogenesis through its influence on pericytes, and our study has answered this question. pNGF as the co-delivery gene of pVEGF enhanced the recruitment of pericytes and strengthened the stability of neovascularization in 28 days. Notably, compared with pVEGF, co-delivery of pNGF in ischemic hindlimbs promoted pericyte recruitment. In future studies, using gene therapy to promote the recruitment and coverage of pericytes may be an effective way to promote stable angiogenesis.

For stable angiogenesis, protective gene delivery vector for genes is equally important. In this study, we synthesized 6 s-PLGA-Po-PEG which eliminated H_2_O_2_. NPs based on 6 s-PLGA-Po-PEG protected genes from oxidative damage and improved the microenvironment of cells by decreasing H_2_O_2_. In this study, the removal of H_2_O_2_ from the microenvironment by NPs was not complete, and it was still difficult to restore the lower limb steady-state microenvironment. In future, carrier with the ability to regulate H_2_O_2_ in microenvironment should be explored.

Co-therapy of pVEGF with a second gene has been studied by many researchers to promote stable angiogenesis. Through the co-delivery of pNGF and pVEGF, Bin Gao et al. found that gene expressions of double genome group were significantly increased at mRNA and protein levels, which was at least 2 and 1.5 times of that in single genome group respectively. Moreover, they confirmed that co-delivery of two genes had a synergistic effect, which enhanced the proliferation, migration and angiogenesis of HUVECs significantly [41]. Aiki Marushima et al. achieved better hemodynamic recovery and ischemic protection through the co-delivery of VEGF and PDGF-BB, and increased collateral blood flow of chronic hypoperfusion [42]. Our study proved that, co-delivery of pVEGF and pNGF had better therapeutic efficiency on the stable angiogenesis of ischemic hindlimb. In numerous studies, co-therapy of pVEGF and a second gene had achieved "1 + 1 > 2" therapeutic effect. In the future, combination of polygene therapy and microenvironment improvement may enable gene NPs more functions, which is expected to become a promising way in PVD treatment.

## Conclusion

In summary, we used H_2_O_2_ responsive 6 s-PLGA-Po-PEG to co-loading pVEGF and pNGF and prepared VEGF/NGF-NPs. VEGF/NGF-NPs eliminated H_2_O_2_ while co-delivering pVEGF and pNGF, promoted stable neovascularization in ischemic hindlimbs successfully by strengthening cell interactions between nerves and blood vessels. Therefore, co-gene delivering NPs that simultaneously enhance neurovascular interactions and improve H_2_O_2_ microenvironment may be a promising strategy to achieve stable angiogenesis in PVD treatment.

## Supplementary Information

**Additional file 1**: **Figure S1.** Particle size and potential of NPs. (A) The particles size and zeta potential of Blank-NPs, VEGF-NPs, NGF-NPs and VEGF/NGF-NPs. **Figure S2.** Schematic diagram of NGF-GFP plasmid and VEGF-RFP plasmid. Schematic diagram of NGF-GFP plasmid (A) and VEGF-RFP plasmid (B) used for gene transfection. **Figure S3.** Effects of VEGF/NGF-NPs on cellular interactions through transwell method. (A) Migration of HUVECs after 48 h co-culture with SH-SY5Ys and VEGF/NGF-NPs. (B) Migration of HBVPs after 48 h co-culture with HUVECs and VEGF/NGF-NPs.

## Data Availability

The data that support the findings of this study are available from the corresponding author upon reasonable request.
