# Supplementary material for: H2O2-responsive VEGF/NGF gene co-delivery nano-system achieves stable vascularization in ischemic hindlimbs
Source: J Nanobiotechnology. 2022 Mar 19;20:145. doi: 10.1186/s12951-022-01328-6 (PMC8934504; doi:10.1186/s12951-022-01328-6)
Supplement: Supplementary file 1 — Additional file 1: Figure S1. Particle size and potential of NPs. (A) The particles size and zeta potential of Blank-NPs, VEGF-NPs, NGF-NPs and VEGF/NGF-NPs. Figure S2. Schematic diagram of NGF-GFP plasmid and VEGF-RFP plasmid. Schematic diagram of NGF-GFP plasmid (A) and VEGF-RFP plasmid (B) used for gene transfection. Figure S3. Effects of VEGF/NGF-NPs on cellular interactions through transwell method. (A) Migration of HUVECs after 48 h co-culture with SH-SY5Ys and VEGF/NGF-NPs. (B) Migration of HBVPs after 48 h co-culture with HUVECs and VEGF/NGF-NPs. [file 12951_2022_1328_MOESM1_ESM.docx]

**Additional material**


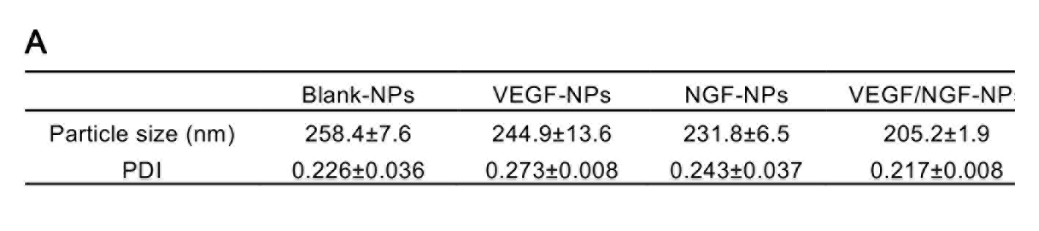


Figure S1. Particle size and potential of NPs. (A) The particles size and zeta potential of Blank-NPs, VEGF-NPs, NGF-NPs and VEGF/NGF-NPs.


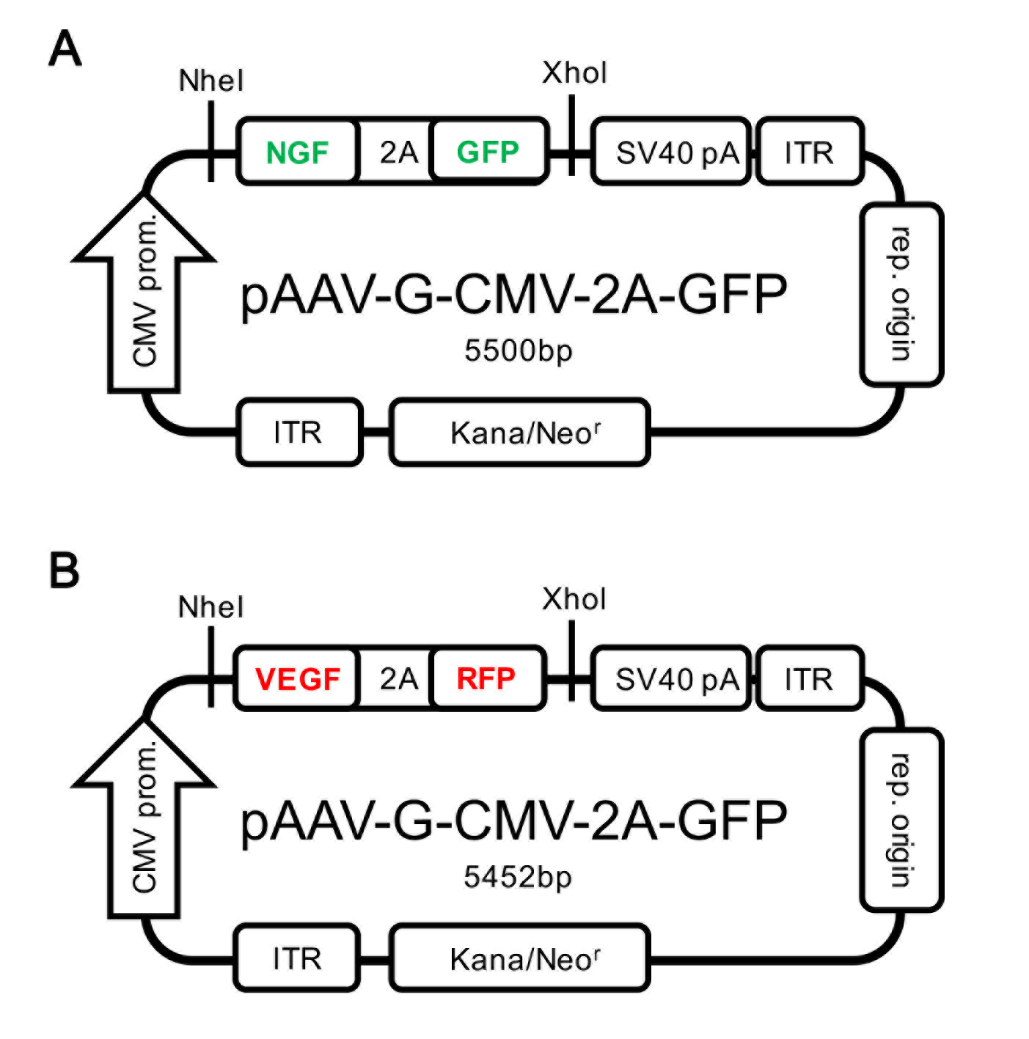


Figure S2. Schematic diagram of NGF-GFP plasmid and VEGF-RFP plasmid. Schematic diagram of NGF-GFP plasmid (A) and VEGF-RFP plasmid (B) used for gene transfection.


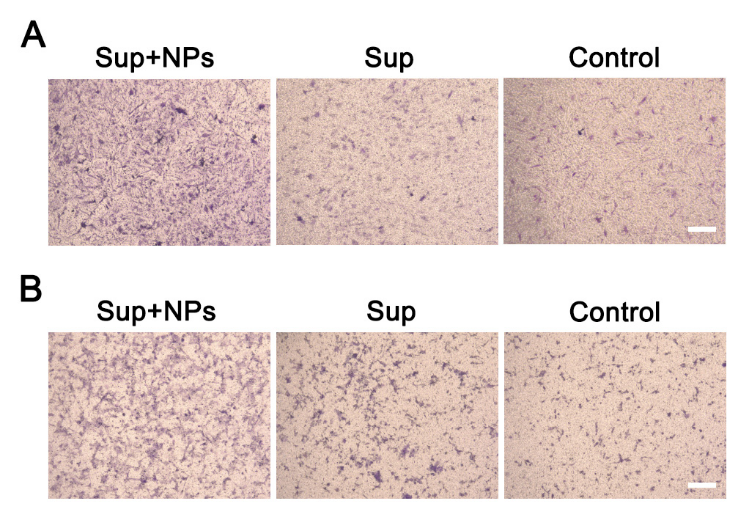


Figure S3. Effects of VEGF/NGF-NPs on cellular interactions through transwell method. (A) Migration of HUVECs after 48 h co-culture with SH-SY5Ys and VEGF/NGF-NPs. (B) Migration of HBVPs after 48 h co-culture with HUVECs and VEGF/NGF-NPs.
